# Replicated Risk Nicotinic Cholinergic Receptor Genes for Nicotine Dependence

**DOI:** 10.3390/genes7110095

**Published:** 2016-11-07

**Authors:** Lingjun Zuo, Rolando Garcia-Milian, Xiaoyun Guo, Chunlong Zhong, Yunlong Tan, Zhiren Wang, Jijun Wang, Xiaoping Wang, Longli Kang, Lu Lu, Xiangning Chen, Chiang-Shan R. Li, Xingguang Luo

**Affiliations:** 1 Department of Psychiatry, Yale University School of Medicine, New Haven, CT 06510, USA; Lingjun.Zuo@yale.edu (L.Z.); Chiang-Shan.Li@yale.edu (C.-S.R.L.); 2 Curriculum & Research Support Department, Cushing/Whitney Medical Library, Yale University School of Medicine, New Haven, CT 06510, USA; Rolando.Milian@yale.edu; 3 Shanghai Mental Health Center, Shanghai 200030, China; jijunwang27@163.com; 4 Department of Cellular and Molecular Physiology, Yale University School of Medicine, New Haven, CT 06510, USA; 5 Department of Neurosurgery, Ren Ji Hospital, School of Medicine, Shanghai Jiao Tong University, Shanghai 200127, China; 6 Biological Psychiatry Research Center, Beijing Huilongguan Hospital, Beijing 100096, China; yltan21@126.com (Y.T.); zhiren75@163.com (Z.W.); 7 Department of Neurology, Shanghai First People’s Hospital, Shanghai Jiao Tong University, Shanghai 200080, China; x_p_wang@sjtu.edu.cn; 8 Key Laboratory for Molecular Genetic Mechanisms and Intervention Research on High Altitude Diseases of Tibet Autonomous Region, Xizang Minzu University School of Medicine, Xianyang, Shanxi 712082, China; klonglister@gmail.com; 9 Provincial Key Laboratory for Inflammation and Molecular Drug Target, Medical College of Nantong University, Nantong 226001, China; 10 Departments of Genetics, Genomics, Informatics, Anatomy and Neurobiology, University of Tennessee Health Science Center, Memphis, TN 38163, USA; lulu@uthsc.edu; 11 Nevada Institute of Personalized Medicine, University of Nevada, Las Vegas, NV 89154, USA; xiangning.chen@unlv.edu; 12 Department of Psychology, University of Nevada, Las Vegas, NV 89154, USA

**Keywords:** *CHRN*, nAChR, nicotine dependence, replication, bioinformatics

## Abstract

It has been hypothesized that the nicotinic acetylcholine receptors (nAChRs) play important roles in nicotine dependence (ND) and influence the number of cigarettes smoked per day (CPD) in smokers. We compiled the associations between nicotinic cholinergic receptor genes (*CHRNs*) and ND/CPD that were replicated across different studies, reviewed the expression of these risk genes in human/mouse brains, and verified their expression using independent samples of both human and mouse brains. The potential functions of the replicated risk variants were examined using *cis*-eQTL analysis or predicted using a series of bioinformatics analyses. We found replicated and significant associations for ND/CPD at 19 SNPs in six genes in three genomic regions (*CHRNB3-A6*, *CHRNA5-A3-B4* and *CHRNA4*). These six risk genes are expressed in at least 18 distinct areas of the human/mouse brain, with verification in our independent human and mouse brain samples. The risk variants might influence the transcription, expression and splicing of the risk genes, alter RNA secondary or protein structure. We conclude that the replicated associations between *CHRNB3-A6*, *CHRNA5-A3-B4,* *CHRNA4* and ND/CPD are very robust. More research is needed to examine how these genetic variants contribute to the risk for ND/CPD.

## 1. Introduction

Nicotine dependence (ND) is commonly assessed for cigarette smokers with DSM-IV criteria or a severity scale such as the Fagerstrom Test for Nicotine Dependence (FTND) [1]. FTND assesses the frequency of smoking, the number of cigarettes smoked and the urgency to smoke, and is widely used to index the severity of ND. Of the six questions assessed in FTND, the number of cigarettes smoked per day (CPD) has been shown to carry the highest genetic loading [2]. It has been hypothesized that the nicotinic acetylcholine receptors (nAChRs) play important roles in the development of ND and shows a strong association to CPD. The nAChR is named because its endogenous agonist is acetylcholine and the plant alkaloid nicotine also binds to these receptors. Neuronal nAChR include α2–α10 and β2–β4 subunits that are encoded by *CHRNAs* 2–10 and *CHRNBs* 2–4, respectively, whereas muscle-type nAChRs include α1, β1, γ, δ and ε subunits that are encoded by *CHRNA*1, *CHRNB*1, *CHRNG*, *CHRND* and *CHRNE*, respectively (reviewed by Zuo et al. [3]).

In this article, we reviewed the relationship between *CHRNs* and ND or CPD that were replicated across studies. We show that most significant risk variants (84%) for ND/CPD at the *CHRNs* are typically located in non-coding regions, and 95% of them have no direct effects on protein structure (see below). These non-coding genetic variants may have effects on the function of genes by altering the transcription, splicing or stability of the coding mRNAs. The association signals detected from the non-coding regions might be related to the roles of non-coding RNAs (ncRNAs) existing within, or proximate to, these regions, and thus these ncRNAs were explored in this study.

ncRNAs include long non-coding RNAs (LncRNAs) and small non-coding RNAs such as miRNAs, piRNAs, siRNAs, snoRNAs and rasiRNAs. Recent evidence suggests that LncRNAs are involved in a wide variety of cellular functions, including epigenetic silencing, transcriptional regulation, RNA processing and modification [4,5,6]; LncRNAs are also implicated in neural plasticity [7], neuropathological process [8], neurotransmission [9], and stress response [7]. Dysregulation of many LncRNAs has been found to contribute to substance use disorders including alcohol, nicotine, heroin and cocaine dependence. For example, *NEAT2*, an LncRNA regulating synapse formation [10], was up-regulated in alcoholics’ brains [11]; *NEAT2,* *NEAT1,* *MIAT* and *MEG3* were up-regulated in the nucleus accumbens (NAc) of heroin abusers [12]; and *NEAT2, MIAT, MEG3* and *EMX2OS* were elevated in the NAc of cocaine abusers [12]. Smokers had dramatically elevated *H19* expression in airway epithelium [13]; demethylation of *H19* was correlated to chronic alcohol use in men [14]; and many LncRNAs mediated cocaine-induced neural plasticity in the NAc and conferred risk for cocaine dependence [8]. Together, evidence accumulates to support the hypothesis that LncRNAs contribute to the severity of ND, including the number of cigarettes smoked per day (CPD).

In addition to LncRNAs, piRNAs are also increasingly being studied for their roles in cellular functions. Numerous research indicates that piRNAs have important roles in modulating mRNA stability, regulating target mRNAs and translation [15], preserving genomic integrity [16], suppressing transposons [17], remodelling euchromatin, developmental regulation and epigenetic programming [18,19]. Recent evidence suggests that piRNAs are abundant in the brain [17,20,21,22,23,24,25,26,27]. These piRNAs have unique biogenesis patterns and are associated with a neuronal Piwi protein. Thus, it has been hypothesized that piRNAs may potentially play roles in ND/CPD too. The LncRNAs and piRNAs that might regulate the effects of the replicated risk *CHRNs* on disease were analyzed in this study. This analysis is a necessary step towards identification of the missing regulatory pathways after a long history of attention to the coding mRNAs and other ncRNAs such as miRNAs.

In this article, we also reviewed the distribution of the nAChRs encoded by the replicated risk *CHRNs* in the human/mouse brain and then verified their expression in an independent sample of mouse brain. Furthermore, we explored the possible mechanisms underlying these replicated associations using a series of bioinformatics analyses.

## 2. Materials and Methods

### 2.1. The Replicated Associations between Nicotinic Cholinergic Receptor Genes (CHRNs) and Nicotine Dependence/Cigarettes per Day (ND/CPD) and the Expression of Risk Genes in Brain

In PubMed (http://www.ncbi.nlm.nih.gov/pubmed), we searched for the literature using the keywords “(nicotinic acetylcholine receptor OR nAChR OR nicotinic cholinergic receptor OR CHRN) AND (nicotine dependence OR nicotine addiction OR smoking OR cigarette)” and obtained 2463 reports (as of 19 September 2016). From these articles, we extracted the established associations between *CHRNs* and ND/CPD. We noticed that although most of the distinct *CHRNs* have been associated with ND/CPD, the replicable associations at single-point level by different studies are rare. We list such rare associations for six genes in three genomic regions from a total of 20 studies in Table 1.

**Figure 1:**
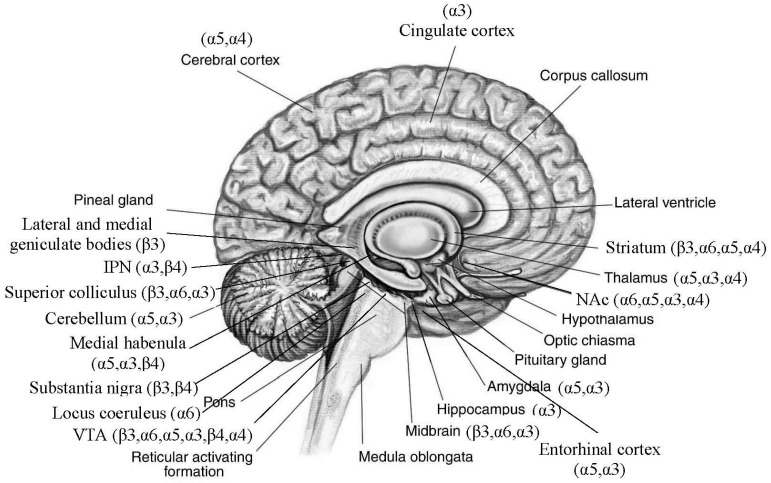
Distribution of nAChR subunits in brain.

Additionally, the distribution of the nAChRs encoded by the replicated risk *CHRNs* reported in the literature is illustrated in Figure 1 (http://anatomy-bodychart.us/) [28,29,30,31,32,33,34,35,36,37,38,39,40,41,42,43,44,45,46,47,48,49,50,51,52,53].

### 2.2. Expression Correlation Analysis in Human Brain

Based on our review (Figure 1), all six replicated risk *CHRNs* are expressed in the midbrain that is enriched with dopaminergic neurons, and four *CHRNs* (i.e., *CHRNA4*, *CHRNA5, CHRNA6* and *CHRNB3*) are expressed in the striatum that is enriched with GABAergic terminals. These are two main neurotransmission systems that have been related to *CHRNs* in the literature (see Section 4: Discussion). We evaluated the mRNA expression levels of these genes and the dopaminergic and GABAergic receptors/enzymes in two independent brain tissue samples using Affymetrix Human ST 1.0 exon arrays (validated by qPCR). The first sample included ten human brain tissues extracted from 134 Europeans (UK Brain Expression Consortium (UKBEC) [74]). These 134 individuals were free of neurodegenerative disorders, and the ten brain tissues included cerebellar cortex, frontal cortex, temporal cortex, occipital cortex, putamen, thalamus, hippocampus, substantia nigra, intralobular white matter and medulla. The second sample included 93 autopsy-collected human frontal cortical tissues [75]. These 93 individuals included 55 male and 38 female Europeans, from 34 to 104 years old with an average of 74 ± 16 years. The postmortem intervals, i.e., the time from death to brain tissue collection, were 1.2–46 h with an average of 14.3 ± 9.5 h. These 93 individuals had no defined neuropsychiatric condition either. Correlations between expression of the risk *CHRNs* and expression of 25 dopaminergic and GABAergic receptor/enzyme genes were tested using Pearson correlation analysis for the first sample and generalized linear model (GLM) analysis for the second sample (Table 2). The 25 dopaminergic and GABAergic genes were *DRD1-5, TH, GABRA1-6, GABRB1-3, GABRD, GABRE, GABRG1-3, GABRR1-3, GABRP* and *GABRQ*. In the GLM, the expression levels of *CHRNs* served as dependent variable, and those of dopaminergic and GABAergic receptor genes as independent variable, by correcting for age, sex and postmortem interval. The directions of the correlations will be shown by the signs of correlation coefficients (r) or regression coefficients (β) (Supplementary Tables S1 and S2). α was set at 3.5 × 10^−5^ for the first sample because 10 brain regions, 25 dopaminergic and GABAergic genes and six *CHRNs* were evaluated, 6.9 × 10^−7^ for the second sample because 12,114 transcripts in the array and six *CHRNs* were evaluated.

### 2.3. Detection of Chrn mRNA Expression in Mouse Brains

To verify the expression of the six replicated risk genes (Figure 1), we examined their mRNA expression in mouse brains in our own samples. The levels of mRNA expression for the whole brain and in eight brain areas were examined, including the cortex, dorsal striatum, NAc, hippocampus, amygdala, midbrain, ventral tegmental area (VTA) and cerebellum (Table 3). The details for mouse strains, gene expression analysis, and calculation for standardized expression values (SEVs) and fold changes (FCs) were published previously [3].

### 2.4. Cis-Acting Genetic Regulation of Expression Analysis in Human Brain Tissues

To examine relationships between the replicated risk *CHRN* variants and local *CHRN* mRNA expression levels, we performed *cis*-acting expression of quantitative locus (*cis*-eQTL) analysis. Expression and genotype data of the six replicated risk *CHRN* genes in ten human brain tissues of the above first sample (i.e., 134 Europeans [74]) were evaluated. Differences in the distribution of mRNA expression levels between SNP genotypes were compared using a Wilcoxon-type trend test. *p*-values less than 0.05 were listed in Table 4. Significance level (α) was corrected by the numbers of tissues, genes and haplotype blocks, i.e., α = 2.8 × 10^−4^ = 0.05/(10 brain tissues × 6 genes × 3 independent haplotype blocks where the 19 replicated SNPs were located).

### 2.5. Bioinformatics Analysis

The linkage disequilibrium (LD) between the replicated risk SNPs was assessed using online HapMap data. To verify the potential functions of these replicated risk SNPs, we predicted their functions using a series of bioinformatics analyses. We used UCSC Genome Browser data or other bioinformatics analysis software packages (e.g., FuncPred [76] or VE!P [77]) to see whether the risk SNPs are located in LncRNAs, in transcription factor binding sites (TFBS), in open chromatin regions, within methylated CpG islands, within copy number variations (CNVs) or in exonic splicing silencers (ESS) or enhancers (ESE). Additionally, Polyphen [78] and SIFT [79] were applied to predict the pathogenicity in order to see whether these risk SNPs affect protein function or structure, and MFOLD [80] was applied to predict whether these risk SNPs alter secondary RNA structure. The conservation of these risk SNPs across 17 species was also predicted [81]. The tertiary structure of the mutant and wild-type protein obtained by translation of each mutant gene was simulated using SWISS-MODEL software [82] so as to find the difference between them.

### 2.6. Long Non-Coding RNAs (LncRNA) and piRNA Analysis

There are tens of thousands of LncRNAs (>200 nt) across the transcriptome [5,83,84], and more than half of them are expressed in the brain [85]. According to the positional relationship between LncRNAs and their associated protein-coding genes, LncRNAs can be classified as intergenic, intronic, antisense, sense overlapping, and bidirectional lncRNAs [86]. In this study, we extracted the LncRNAs close to, or within, the risk *CHRN* genes from the National Center for Biotechnology Information (NCBI) Gene database (http://www.ncbi.nlm.nih.gov/gene).

The RNAs interacting with the Piwi subfamily of proteins in Piwi/piRNA complex are named piRNAs. piRNAs are a class of small ncRNAs originally isolated from the mammalian germline, but recently they have also been detected in the brain [21,22,24]. Each species usually has hundreds of thousands of unique piRNA sequences. Mature piRNAs are short, single-stranded RNA molecules approximately 24–32 nucleotides in length. They are unevenly distributed in the genome, and usually cluster in some specific genomic loci. In this article, we searched for piRNAs within the risk *CHRN* genes from the piRNABank database [87].

## 3. Results

### 3.1. Replicated Associations between CHRNs and ND/CPD (Table 1)

Replicated associations for ND/CPD were found at 19 SNPs in three genomic regions (*CHRNB3-A6*, *CHRNA5-A3-B4* and *CHRNA4*) in Europeans, Africans and Asians. They were replicated across at least three independent samples in at least two independent studies including genome-wide association studies (GWASs) [54,62,66,68,69,71,88] and candidate gene studies [55,56,57,58,59,60,61,63,64,67], and some of them were verified by functional studies.

The associations for *CHRNA5-A3-B4* were most comprehensively studied and most robust; many of them were highly significant with *p* values below 10^−72^; and many of them were detected by high-impact unbiased GWASs. For example, Thorgeirsson et al. [71] (2008) and Liu et al. [68] (2010) reported associations between rs1051730 at *CHRNA*3 and smoking quantity (*p* = 5 × 10^−16^ and 1.7 × 10^−66^, respectively). This association has been replicated by numerous other GWASs [89,90,91] and candidate gene studies [56,72,92] and in a meta-analysis (*p* = 2.75 × 10^−73^ in the subjects of European ancestry) [57,62,66,68]. Liu et al. [68] (2010) also reported associations between rs16969968 at *CHRNA*5 (*p* = 4.3 × 10^−65^) and rs6495308 at *CHRNA*3 (*p* = 5.8 × 10^−44^) and smoking quantity. These two SNPs were also associated with ND [92,93]. rs16969968 was a non-synonymous, functional SNP [88] and was associated with experiencing pleasurable response upon first-time smoking, with current smoking status [94] and with ND [56,61,93], which was supported by some meta-analyses (*p* = 5.57 × 10^−72^ in European) [57,62,66,68]. Berrettini et al. [72] (2008) also reported in a GWAS that rs6495308 at *CHRNA*3 was associated with CPD (*p* = 6.9 × 10^−5^). Additionally, a common haplotype at *CHRNA*5 and *CHRNA*3 increased risk across a series of ND-related phenotypes among European-origin populations, including ND [88,89,91,92,95], early-onset ND [96,97], CPD [98], FTND score [89,96], inability to quit when pregnant [99], serum cotinine (a nicotine metabolite) level [95,100], and chronic obstructive pulmonary disease [101]. Finally, rare variant analysis showed that rare missense variants at conserved residues in *CHRNB*4 were associated with reduced risk of ND among African Americans [102]. Among these studies, at least five studies that identified peak SNPs rs16969968 and rs1051730 at *CHRNA5-A3-B4* as risk markers for ND or CPD were high-impact GWASs (Table 1) [62,66,68,69,71,88].

Additionally, several other GWASs identified association peak for ND at *CHRNA*4 [64,69,89], a finding that has been widely replicated [56,57,64,89,103,104]. Many other GWASs also showed an association of *CHRNB3–CHRNA6* with nicotine addiction [62,89,92,105], which was replicated by many other candidate gene studies [56,57,58,60,64,65]. This region was also associated with subjective response to tobacco use [106].

### 3.2. Distributions of Nicotinic Acetylcholine Receptors (nAChRs) Encoded by the Replicated Risk CHRNs in Brain (Figure 1)

The three replicated genomic regions including six genes are expressed in at least 18 brain areas. They are most commonly expressed in medial habenula, midbrain (including the VTA, substantia nigra, interpeduncular nucleus (IPN), lateral and medial geniculate bodies, and superior colliculus) and the mesolimbic system (VTA→NAc). They are also expressed in cortex, entorhinal cortex, striatum, thalamus, hippocampus, amygdala, locus coeruleus, brainstem nuclei and cerebellum. Specifically, *CHRNA5, CHRNA3* and *CHRNB4* are highly expressed in medial habenula. All six genes are expressed in the midbrain, although different genes have distinct densities in different midbrain areas. *CHRNA4* is expressed in the thalamus at the highest level. *CHRNA3* and *CHRNA5* are also expressed in the thalamus, with *α5* in low density. *CHRNA5* and *CHRNA3* are expressed in or around the hippocampus. Both have expression in amygdala and entorhinal cortex. *CHRNA3* also has a low level of expression in hippocampus. *CHRNA5* and *CHRNA4* are expressed in cortex. *CHRNA3* is also expressed in cingulate cortex and insular cortex at low density. *CHRNA5, CHRNA4, CHRNA6* and *CHRNB3* are expressed in striatum. *CHRNA6* is expressed in locus coeruleus, a noradrenergic nucleus with wide projections to cortical and subcortical structures [107]. *CHRNA3* is also expressed in brainstem nuclei. Finally, *CHRNA5* and *CHRNA3* are expressed in cerebellum.

### 3.3. All Six CHRN Genes Were Expressed in Human Brain and Their Expression Was Correlated with Dopaminergic or GABAergic Expression (Table 2, Tables S1 and S2)

These six *CHRN* genes were detected in ten human brain areas. In many areas, their expression was significantly correlated with the dopaminergic or GABAergic expression (*p* < α) (Table 2). The correlation coefficients (0.358 ≤ |r| ≤ 0.920), regression coefficients (0.008 ≤ |β| ≤ 0.749) and *p* values (3.9 × 10^−42^ ≤ *p* ≤ 3.3 × 10^−5^) for these correlations are shown in the Supplementary Tables S1 and S2.

### 3.4. All Six Chrn Genes Were Expressed in Mouse Brain in Distinct Areas and at Different Levels, a Majority of Which Verified Previous Reports (Table 3)

We found that all six *Chrn* genes were expressed in mouse brain at different levels. All of these genes were expressed in the hippocampus, in which the gene with the most highly abundant expression (SEV > 9) was *Chrna4* (SEV = 9.95). α4 mRNA was also abundant in other brain areas examined (SEV = 8.29–10.73), with 2.5-13.3-FCs in mRNA level compared to the expression base. Compared with other genes, α4 mRNA was also the most abundant in the whole brain and five other brain areas including cortex, striatum, NAc, amygdala and cerebellum (Table 3).

*Chrna5*, *Chrna3* and *Chrnb4* were expressed in multiple brain areas (SEV = 7.22–9.35), with a 1.2-5.1-FC in mRNA expression levels compared to the expression base (SEV = 7). *Chrna6* and *Chrnb3* were expressed in several areas (SEV = 7.11–10.37), among which a6 mRNA was the most abundant in the midbrain (FC = 10.4) and VTA (FC = 3.9) among all six *Chrns*. Many of these findings verified the previous reports described above.

### 3.5. The CHRN Variants May Regulate the Expression of CHRN Genes (Table 4)

*Cis-*eQTL analysis showed that 13 risk SNPs at *CHRNB3-CHRNA6* had nominally significant *cis-*acting regulatory effects on *CHRNB3* mRNA expression in cerebellar cortex and thalamus (*p* = 0.015–0.022 and 0.026–0.031, respectively), and on *CHRNA6* mRNA expression in frontal cortex and hippocampus (*p* = 0.042–0.043 and 0.027–0.033, respectively). Three risk SNPs at *CHRNA5-CHRNA3-CHRNB4* had nominally significant *cis-*acting regulatory effects on *CHRNA5* mRNA expression in almost all ten brain areas (5.1 × 10^−6^ ≤ *p* ≤ 0.034), and on *CHRNA3* mRNA expression in putamen (8.9 × 10^−4^ ≤ *p* ≤2.7 × 10^−3^). rs6495308 at this region also had nominally significant *cis*-acting regulatory effects on *CHRNB4* mRNA expression in occipital cortex and medulla (0.014 ≤ *p* ≤ 0.025). rs2236196 at *CHRNA4* had nominally significant *cis-*acting regulatory effects on *CHRNA4* mRNA expression in intralobular white matter and medulla (0.035 ≤ *p* ≤ 0.044). After Bonferroni correction (α = 2.8 × 10^−4^), the regulatory effects of the three risk SNPs at *CHRNA5-CHRNA3-CHRNB4* on *CHRNA5* mRNA expression remained significant in seven brain areas.

### 3.6. Bioinformatics Analysis (Table 5)

Of the 19 replicated risk variants, 15 SNPs at *CHRNB3-CHRNA6*, three SNPs at *CHRNA5-CHRNA3-CHRNB4*, and one SNP at *CHRNA4* are included. The 15 SNPs at *CHRNB3-CHRNA6* are all in high LD (D’ > 0.95) (https://hapmap.ncbi.nlm.nih.gov/). Among the 19 risk SNPs, 10 SNPs are located in LncRNAs that might regulate the gene expression. There are eight SNPs located in the TFBS. Most of them are located in the 5′ to *CHRNB3*. They may affect the local DNA conformation, and thereby influence the binding of transcription factors [108]. Two SNPs, i.e., rs4954 and rs2236196, are located in the open chromatin regions, which are often associated with regulatory factor binding. One SNP, rs1051730 at the exon 7 of *CHRNA3*, is located within a 234 bp CpG island, whose methylation status may affect the expression of *CHRNA3* [109]. rs16969968 (Asp398Asn) at the exon 5 of *CHRNA5* is located in an exonic splicing silencer or enhancer. Furthermore, seven SNPs are predicted to significantly or highly significantly alter the RNA secondary structures, including rs10958725, rs13273442, rs4736835, rs13277524, rs6474412 and rs4952 at *CHRNB3*, and rs16969968 at *CHRNA5*. Two SNPs are predicted to mildly alter the RNA secondary structures, including rs1955186 and rs7004381 at *CHRNB3*. They may affect the downstream activities of the RNA molecules [110]. rs16969968 is also predicted to be conservative across species. Finally, amino acid sequence alignment and three-dimensional computer space model verify that rs16969968 highly significantly alters protein structure and function (Supplementary Figure S1).

### 3.7. The LncRNAs and piRNAs Related to the Replicated Risk CHRNs

The LncRNAs proximate to each gene are listed in Table 6. One sense LncRNA 37 kb to *CHRNB4* is a large intergenic non-coding RNA (LincRNA), with a length of 35 kb; two others overlapping with *CHRNB3* and *CHRNA4* are antisense LncRNAs, with lengths of 11 kb to 22 kb. The annotated piRNAs mapping within the two replicated *CHRN* gene regions are listed in Table 7. These piRNAs show a size distribution between 26 and 31 nt.

## 4. Discussion

Replicated associations for ND/CPD were found at 19 SNPs in three genomic regions (*CHRNB3-A6*, *CHRNA5-A3-B4* and *CHRNA4*). Many of these associations are highly replicable across studies, highly significant, verified by functional studies, and supported by bioinformatics analysis, and thus are very robust. Interestingly, these three replicated loci were just the top three peak risk loci for ND identified by a GWAS meta-analysis using a large sample size of 17,074 [69]. We believe that *CHRNB3-A6*, *CHRNA5-A3-B4* and *CHRNA4* play important roles in the susceptibility to ND/CPD. Mechanisms underlying these roles may be related to the brain areas where the risk genes are expressed, the specific functions of the risk variants, or the regulatory pathways for the expression of these risk genes.

All replicated risk genes were expressed in human/mouse brain regions, which was verified at the mRNA level in our independent samples of both human and mouse brains. Many of these brain areas are important for the development of drug dependence [111]. Functional data have shown changes in nicotine intake following manipulations of α5*, α3* and β4* nAChRs in the medial habenula, supporting that medial habenula could contribute to the reinforcing effect of nicotine [28]. Many areas in midbrain are enriched in dopaminergic neurons, including VTA (where all six replicated risk genes were expressed) and substantia nigra (β3* and β4*). We demonstrated that the mRNA expression of six *CHRNs* was correlated with the expression of dopaminergic receptor/enzyme genes in ten brain areas. Thus, the *CHRN* receptors in these areas may modulate dopamine release, and contribute to the reinforcing effect of nicotine. Several pathways in the midbrain, e.g., habenula-IPN pathway (α5*, α3* and β4*) and VTA-NAc pathway (i.e., mesolimbic system; α5*, α3* and α4*), are also critical to drug-induced reward responses. The thalamus plays a major role in relaying and transforming information to the cortex and in turn modulates cortical outputs. Imaging studies in humans implicated the thalamus in cognitive control [112], a process frequently compromised in individuals with addiction [113]. Nicotine binding to α4* nAChRs in the human thalamus is very high in most thalamic nuclei, especially in the lateral dorsal, the medial geniculate, lateral geniculate and anterior nuclei. Striatum (α5*, α4*, α6* and β3*) receives dopaminergic input to the GABAergic medium spiny neurons. We demonstrated that the mRNA expression of six *CHRNs* was correlated with the expression of GABA receptor genes, supporting that nicotine stimulation of dopamine and GABA terminals in striatum may facilitate the release of these neurotransmitters. The locus coeruleus (α6*) is the principal site for brain synthesis of norepinephrine (noradrenaline). This nucleus may be involved in physiological responses to stress and panic, and some symptoms of ND. Finally, the hippocampus, amygdala, cortex including entorhinal cortex, and cerebellum are involved in reward, learning, motor co-ordination, memory and/or emotion. Nicotine may direct information flow through the neural circuits via the activation of α5*, α4* and α3* in these areas.

Our *cis*-eQTL and bioinformatics analyses provided additional evidence to support the previous findings that these replicated risk SNPs were functional [114,115,116]. They might influence the transcription, expression and splicing of the risk genes; they might alter the RNA secondary structure and thus affect the downstream activities of the RNA molecules; or they might even alter the structure and function of the proteins encoded by these risk genes. This analysis supports the roles of *CHRNs* in ND/CPD.

The sense LincRNA usually collaborates with chromatin modifying proteins (PRC2, CoREST and SCMX) to regulate expression of proximate genes [117]. Accordingly, we can postulate that the LincRNA XR_932509.1 might potentially regulate the expression of the *CHRNB4* and might be functional components of the pathways through which the *CHRNB4* variants influence risk for ND/CPD. The other two antisense-overlapping LncRNAs, i.e., XR_949716.1 and NR_110634.1, might use diverse transcriptional and post-transcriptional mechanisms [118,119] to regulate *CHRNB3* and *CHRNA4* to play roles in ND/CPD.

The piRNAs in brain usually show unique biogenesis patterns and predominantly nuclear localization [20]. The influence of piRNAs on disease might depend on the neurotransmitters/genes they interact with or the brain areas they are expressed in. For example, the piRNAs may have robust sensitivity to serotonin, a neurotransmitter with important roles in learning and memory and widely implicated in the etiology of many mental disorders [18,20]. The Piwi/piRNA complex may facilitate serotonin-dependent methylation of a conserved CpG island in the promoter of *CREB2*, the major inhibitory constraint of memory, leading to enhanced long-term learning-related synaptic facilitation [20]. Some piRNAs expressed in hippocampal neurons may influence dendritic spine morphogenesis [21]. For instance, piRNAs may target Astrotactin, which has been implicated in neuronal migration [120] or regulate genes to control nervous system function [21]. One is tempted to speculate that these piRNAs might potentially regulate the expression of the risk genes and serve as functional components of the pathways through which the risk SNPs influence risk for ND/CPD. These hypotheses regarding LncRNAs and piRNAs should be tested in the future.

## Figures and Tables

**Table 1:** Replicated associations between *CHRN* genes and nicotine dependence.

SNP	Gene	*p*	Ref.	*p*	Ref.	*p*	Ref.	*p*	Ref.	*p*	Ref.	*p*	Ref.	*p*	Ref.	*p*	Ref.
rs10958725	*CHRNB3-A6*	3.1 × 10^−8^	[54]	4.7 × 10^−3^	[55]	3.6 × 10^−5^	[55]										
rs10958726	*CHRNB3-A6*	1.2 × 10^−7^	[54]	9.6 × 10^−5^	[56]	5.7 × 10^−3^	[57]	1.1 × 10^−3^	[57]	1.1 × 10^−2^	[55]	1.4 × 10^−5^	[55]				
rs13273442	*CHRNB3-A6*	1.4 × 10^−7^	[54]	2.0 × 10^−2^	[58]	1.4 × 10^−3^	[58]	3.0 × 10^−2^	[58]								
rs4736835	*CHRNB3-A6*	3.0 × 10^−8^	[54]	6.0 × 10^−3^	[55]	6.2 × 10^−3^	[57]										
rs1955186	*CHRNB3-A6*	8.3 × 10^−5^	[56]	5.4 × 10^−3^	[57]	1.1 × 10^−2^	[57]										
rs1955185	*CHRNB3-A6*	4.6 × 10^−8^	[54]	1.0 × 10^−4^	[56]	1.1 × 10^−5^	[55]	5.4 × 10^−3^	[57]	1.2 × 10^−3^	[57]						
rs13277254	*CHRNB3-A6*	4.0 × 10^−3^	[59]	4.0 × 10^−5^	[56]	7.8 × 10^−4^	[57]	6.3 × 10^−4^	[60]								
rs13277524	*CHRNB3-A6*	6.0 × 10^−5^	[56]	3.8 × 10^−3^	[57]	7.4 × 10^−4^	[57]										
rs6474412	*CHRNB3-A6*	1.1 × 10^−4^	[56]	5.6 × 10^−3^	[57]	1.0 × 10^−3^	[61]	8.7 × 10^−3^	[55]	2.1 × 10^−5^	[55]	* 1.7 × 10^−4^	[62]	* 2.6 × 10^−5^	[62]	* 8.0 × 10^−3^	[63]
rs6474413	*CHRNB3-A6*	3.6 × 10^−8^	[54]	6.3 × 10^−5^	[56]	9.3 × 10^−4^	[57]										
rs7004381	*CHRNB3-A6*	9.9 × 10^−8^	[54]	3.9 × 10^−2^	[60]	3.1 × 10^−3^	[57]										
rs4950	*CHRNB3-A6*	9.5 × 10^−8^	[54]	1.0 × 10^−4^	[56]	1.4 × 10^−3^	[57]	7.0 × 10^−3^	[60]	1.1 × 10^−5^	[55]						
rs13280604	*CHRNB3-A6*	1.0 × 10^−7^	[54]	6.0 × 10^−3^	[60]	1.4 × 10^−5^	[55]	* 1.2 × 10^−4^	[62]	* 2.7 × 10^−5^	[62]						
rs4952	*CHRNB3-A6*	4.1 × 10^−3^	[56]	1.1 × 10^−2^	[57]	1.4 × 10^−3^	[57]	2.0 × 10^−2^	[58]								
rs4954	*CHRNB3-A6*	4.3 × 10^−7^	[64]	6.0 × 10^−3^	[65]	4.1 × 10^−3^	[57]										
rs16969968	*CHRNA5-A3-B4*	1.0 × 10^−2^	[59]	1.3 × 10^−4^	[56]	* 2.4 × 10^−69^	[62]	* 5.6 × 10^−72^	[66]	* 9.0 × 10^−4^	[67]	* 4.3 × 10^−65^	[68]	5.1 × 10^−17^	[69]		
rs1051730	*CHRNA5-A3-B4*	2.0 × 10^−4^	[56]	2.0 × 10^−3^	[70]	* 5.8 × 10^−44^	[68]	* 2.8 × 10^−73^	[66]	* 1.0 × 10^−3^	[67]	* 1.7 × 10^−66^	[68]	* 6.0 × 10^−20^	[71]	4.3 × 10^−17^	[69]
rs6495308	*CHRNA5-A3-B4*	1.9 × 10^−3^	[56]	* 6.9 × 10^−5^	[72]	4.8 × 10^−3^	[56]	1.7 × 10^−7^	[69]								
rs2236196	*CHRNA4*	3.1 × 10^−7^	[64]	2.0 × 10^−2^	[73]	5.0 × 10^−4^	[57]	4.4 × 10^−4^	[57]	2.7 × 10^−2^	[69]						

*p. p-*value; Ref. reference. * associations with cigarettes per day (CPD). The associations identified by GWASs were underlined.

**Table 2:** Significant expression correlation between *CHRNs* and dopaminergic and GABAergic receptor genes in human brain.

Genes	*CHRNB3*	*CHRNA6*	*CHRNA5*	*CHRNA3*	*CHRNB4*	*CHRNA4*
DRD1	SNIG	PUTM,TCTX		FCTX,TCTX		FCTX,THAL
DRD2	SNIG,TCTX	PUTM,SNIG,TCTX,THAL		FCTX,HIPP,TCTX,THAL	CRBL,FCTX	CRBL,OCTX,SNIG,TCTX,THAL
DRD3	FCTX	PUTM	THAL	FCTX	FCTX,OCTX	
DRD4	FCTX		THAL	FCTX,TCTX	FCTX,HIPP,OCTX,PUTM,TCTX	
DRD5	WHMT	THAL	THAL	FCTX,THAL,WHMT	CRBL,FCTX,HIPP,SNIG,WHMT	THAL
TH	SNIG,TCTX,WHMT	SNIG,TCTX		FCTX,TCTX	CRBL,FCTX,OCTX	CRBL,SNIG
GABRA1	SNIG	SNIG,THAL	SNIG,THAL	CRBL,THAL	FCTX	FCTX,HIPP,MEDU,SNIG,THAL
GABRA2	OCTX	MEDU,OCTX,PUTM,THAL	CRBL,MEDU	FCTX,MEDU,TCTX,THAL	FCTX,MEDU	OCTX,THAL
GABRA3	OCTX,SNIG	MEDU,OCTX,SNIG,THAL	MEDU,SNIG,THAL	CRBL,THAL		CRBL,FCTX,HIPP,OCTX,PUTM,SNIG,TCTX,THAL
GABRA4	FCTX,OCTX,SNIG,THAL	MEDU,OCTX,PUTM,SNIG,THAL,WHMT	CRBL,MEDU,THAL	FCTX,MEDU,TCTX,THAL,WHMT	FCTX,MEDU,TCTX	CRBL,FCTX,HIPP,SNIG,THAL
GABRA5	OCTX	MEDU,OCTX,THAL	MEDU,THAL	MEDU,THAL	CRBL	CRBL,FCTX,OCTX,THAL
GABRA6				CRBL		
GABRB1	FCTX,OCTX,PUTM,SNIG	MEDU,PUTM,SNIG,THAL	CRBL,MEDU,SNIG	FCTX,OCTX,TCTX	FCTX,TCTX	OCTX,SNIG
GABRB2	SNIG	SNIG,THAL	CRBL,THAL	CRBL,FCTX,PUTM,TCTX,THAL	FCTX,TCTX	FCTX,HIPP,MEDU,THAL
GABRB3	OCTX,SNIG	MEDU,OCTX,SNIG,THAL	MEDU,THAL	MEDU,TCTX,THAL	MEDU,TCTX	CRBL,FCTX,OCTX,SNIG,THAL
GABRD	WHMT	THAL	THAL	CRBL,PUTM,THAL,WHMT		FCTX,HIPP,MEDU,THAL
GABRE		THAL				MEDU
GABRG1		MEDU	CRBL,MEDU	MEDU,OCTX		
GABRG2	OCTX,SNIG,WHMT	SNIG,THAL	THAL	CRBL,TCTX,THAL	TCTX	FCTX,HIPP,MEDU,SNIG,THAL
GABRG3	FCTX	MEDU,PUTM	CRBL	TCTX	FCTX,TCTX	CRBL,FCTX,MEDU
GABRP	FCTX,PUTM			FCTX	CRBL,FCTX,PUTM	
GABRQ	HIPP	THAL	THAL	HIPP,THAL	CRBL	CRBL,MEDU,PUTM,THAL
GABRR2				THAL		

α = 3.3 × 10^−5^. Cerebellar cortex (CRBL), frontal cortex (FCTX), hippocampus (HIPP), medulla (specifically inferior olivary nucleus, MEDU), occipital cortex (specifically primary visual cortex, OCTX), putamen (PUTM), substantia nigra (SNIG), temporal cortex (TCTX), thalamus (THAL), and intralobular white matter (WHMT). These six CHRN genes were detected in ten human brain areas. In many areas, their expression was significantly correlated with the dopaminergic or GABAergic expression (*p* < α) (Table 2). The correlation coefficients (0.358 ≤ |r| ≤ 0.920), regression coefficients (0.008 ≤ |β| ≤ 0.749) and *p* values (3.9 × 10^−42^ ≤ *p* ≤ 3.3 × 10^−5^) for these correlations are shown in the Supplementary Tables S1 and S2.

**Table 3:** *Chrn* gene expression at whole brain and different brain areas of BXD mice.

Gene	Location (Chr, Mb)	Whole Brain	Cortex	Striatum	NAc	Hippocampus	Amygdala	Midbrain	VTA	Cerebellum
*Chrnb3*	Chr8: 28.504645	7.85	7.11			7.25			7.65	
*Chrna6*	Chr8: 28.513939	8.73			7.79	7.13		10.37	8.97	7.23
*Chrna5*	Chr9: 54.852890		7.22			8.44	7.45			
*Chrna3*	Chr9: 54.860390	9.35			7.22	8.32			7.60	7.61
*Chrnb4*	Chr9: 54.877893	7.23		7.58		8.65	8.18	8.23		
*Chrna4*	Chr2: 180.759407	9.48	10.05	9.02	8.29	9.95	10.73	9.64	8.71	8.30

The order of the gene list corresponds to Table 1. Only the standardized expression values (SEV) > 7 are listed. The expression replicating the previous reports (Figure 1) is underlined. This is a sub-table of the Table 5 in the paper by Zuo et al. [3].

**Table 4:** *Cis*-acting expression of quantitative locus (*cis*-eQTL) analysis.

SNPs	Target gene	Cerebellar Cortex	Frontal Cortex	Temporal Cortex	Occipital Cortex	Putamen	Thalamus	Hippo-Campus	Substantia Nigra	Intralobular White Matter	Medulla
rs10958725	*CHRNB3*	0.015					0.026				
rs10958725	*CHRNA6*		0.042					0.027			
rs10958726	*CHRNB3*	0.020					0.028				
rs10958726	*CHRNA6*		0.043					0.031			
rs13273442	*CHRNB3*	0.022					0.030				
rs13273442	*CHRNA6*		0.043					0.032			
rs4736835	*CHRNB3*	0.022					0.030				
rs4736835	*CHRNA6*		0.043					0.032			
rs1955186	*CHRNB3*	0.022					0.030				
rs1955186	*CHRNA6*		0.043					0.032			
rs1955185	*CHRNB3*	0.022					0.030				
rs1955185	*CHRNA6*		0.043					0.032			
rs13277254	*CHRNB3*	0.021					0.031				
rs13277254	*CHRNA6*		0.042					0.033			
rs13277524	*CHRNB3*	0.022					0.030				
rs13277524	*CHRNA6*		0.043					0.032			
rs6474412	*CHRNB3*	0.022					0.030				
rs6474412	*CHRNA6*		0.043					0.032			
rs6474413	*CHRNB3*	0.022					0.030				
rs6474413	*CHRNA6*		0.043					0.032			
rs7004381	*CHRNB3*	0.022					0.030				
rs7004381	*CHRNA6*		0.043					0.032			
rs4950	*CHRNB3*	0.022					0.030				
rs4950	*CHRNA6*		0.043					0.032			
rs13280604	*CHRNB3*	0.022					0.030				
rs13280604	*CHRNA6*		0.043					0.032			
rs16969968	*CHRNA5*	0.034	2.0 × 10^−4^	9.3 × 10^−5^	5.1 × 10^−6^	1.9 × 10^−3^	2.2 × 10^−3^	5.9 × 10^−5^	1.8 × 10^−5^	0.016	1.6 × 10^−4^
rs16969968	*CHRNA3*					8.9 × 10^−4^					
rs1051730	*CHRNA5*	0.034	2.0 × 10^−4^	9.3 × 10^−5^	5.1 × 10^−6^	1.9 × 10^−3^	2.2 × 10^−3^	5.9 × 10^−5^	1.8 × 10^−5^	0.016	1.6 × 10^−4^
rs1051730	*CHRNA3*					8.9 × 10^−4^					
rs6495308	*CHRNA5*	5.1 × 10^−3^	1.9 × 10^−4^	4.2 × 10^−3^	8.4 × 10^−3^	2.8 × 10^−4^		1.6 × 10^−4^	7.3 × 10^−3^	3.4 × 10^−3^	
rs6495308	*CHRNA3*					2.7 × 10^−3^					0.013
rs6495308	*CHRNB4*				0.025						0.014
rs2236196	*CHRNA4*									0.044	0.035

α = 2.8 × 10^−4^ = 0.05/(10 brain tissues × 6 genes × 3 haplotype blocks); *n* = 134.

**Table 5:** Bioinformatics analyses on replicable risk *CHRN* SNPs.

SNP	Chr	Position	Location	Allele Frequency				2^nd^ RNA Alteration	Bioinformatics
		(Build 37)		Allele	European	African	Asian		
rs10958725	8	42524584	5′ to CHRNB3	G	0.822	0.239	0.792	Highly significant	--
rs10958726	8	42535909	5′ to CHRNB3	T	0.807	0.328	0.816	no	--
rs13273442	8	42544017	5′ to CHRNB3	G	0.825	0.35	0.826	Significant	--
rs4736835	8	42547033	5′ to CHRNB3	C	0.825	0.35	0.826	Significant	LncRNA
rs1955186	8	42549491	5′ to CHRNB3	C	0.833	0.326	0.875	Mild	TFBS, LncRNA
rs1955185	8	42549647	5′ to CHRNB3	T	0.822	0.233	0.836	no	TFBS, LncRNA
rs13277254	8	42549982	5′ to CHRNB3	A	0.833	0.435	0.875	no	TFBS, LncRNA
rs13277524	8	42550057	5′ to CHRNB3	T	0.833	0.326	0.875	Significant	TFBS, LncRNA
rs6474412	8	42550498	5′ to CHRNB3	T	0.81	0.309	0.824	Significant	TFBS, LncRNA
rs6474413	8	42551064	5′ to CHRNB3	T	0.833	0.235	0.875	no	TFBS, LncRNA
rs7004381	8	42551161	5′ to CHRNB3	G	0.825	0.339	0.826	Mild	TFBS, LncRNA
rs4950	8	42552633	5′UTR of CHRNB3	A	0.828	0.182	0.826	no	TFBS, LncRNA
rs13280604	8	42559586	Intron 1 of CHRNB3	A	0.825	0.178	0.826	no	LncRNA
rs4952	8	42587065	Exon 6 of CHRNB3	C	0.983	1	1	Highly significant	--
rs4954	8	42587796	Intron 6 of CHRNB3	A	0.973	0.773	0.885	no	chromatin
rs16969968 (Asp398Asn)	15	78882925	Exon 5 of CHRNA5	G	0.587	1	0.982	Highly significant	splicing,tolerated, benign,conservative
rs1051730	15	78894339	Exon 7 of CHRNA3	G	0.608	0.876	0.982	no	CpG
rs6495308	15	78907656	Intron 6 of CHRNA3	T	0.792	0.661	0.244	no	--
rs2236196	20	61977556	3′UTR of CHRNA4	A	0.744	0.458	0.889	no	chromatin

2nd RNA alteration, the alteration of secondary RNA structure predicted using MFOLD; LncRNA, these SNPs are located in LncRNAs; TFBS, these SNPs are located in the transcription factor binding sites; chromatin, this SNP is located in an open chromatin region; splicing, this SNP is located in an exonic splicing silencer or enhancer; tolerated/benign, these SNPs are predicted by SIFT/Polyphen not to significantly affect protein function or structure; conservative, this SNP is predicted to be conservative; CpG, this SNP is located within a 234 bp methylated CpG island.

**Table 6:** The long non-coding RNAs (LncRNAs) proximate to the three replicable *CHRN* genes.

LncRNA name (NCBI Gene)	Alias	Length (nt)	Distance to risk gene	Category
XR_949716.1	LOC105379396	21,176	Covering *CHRNB3*	antisense LncRNA
XR_932509.1	LOC105370913	35,230	37,240 bp to *CHRNB4*	intergenic sense LincRNA
NR_110634.1	LOC100130587	11,190	Overlap with exon 1 of *CHRNA4*	antisense LncRNA

Intergenic, located between two protein-coding genes and at least 1 kb away from these genes; Sense, LncRNAs are transcribed from the same genomic strand as the protein-coding mRNAs; Antisense, LncRNAs are transcribed from the antisense strand.

**Table 7:** The annotated piRNAs within the two replicable *CHRN* genes.

Replicable genes	Position (Build 37)	Number of piRNAs	Length (nt)
*CHRNB3*	chr8:42552561–42592208	42	26–31
*CHRNA6*	chr8:42607762–42623618	8	29–31
*CHRNA5*	chr15:78857905–78886459	17	28–31
*CHRNA3*	chr15:78887650:78913321	20	26–31
*CHRNB4*	Chr15:78916635:78933586	4	27–29

## Supplementary Materials

The following are available online at www.mdpi.com/2073-4425/7/11/95/s1, Table S1, Significant expression correlation between CHRNs and dopaminergic and GABAergic receptor genes in ten human brain areas; Table S2, Significant expression correlation between CHRNs and dopaminergic and GABAergic receptor genes in human frontal cortex; Figure S1, The tertiary structures of α5 nAChR altered by rs16969968 (D: Asp; N: Asn).

## Abbreviations

The following abbreviations are used in this manuscript:

nAChRs	nicotinic acetylcholine receptors
*CHRNs*	nicotinic cholinergic receptor genes
ND	nicotine dependence
CPD	cigarettes smoked per day
FTND	Fagerstrom Test for Nicotine Dependence
ncRNAs	non-coding RNAs
LncRNAs	long non-coding RNAs
NAc	nucleus accumbens
VTA	ventral tegmental area
