# Supplementary material for: Replicated Risk Nicotinic Cholinergic Receptor Genes for Nicotine Dependence
Source: Genes (Basel). 2016 Nov 7;7(11):95. doi: 10.3390/genes7110095 (PMC5126781; doi:10.3390/genes7110095)
Supplement: Supplementary file 1 [file genes-07-00095-s001.docx]

Supplementary Materials: Replicated Risk Nicotinic Cholinergic Receptor Genes for Nicotine Dependence

Lingjun Zuo, Rolando Garcia-Milian, Xiaoyun Guo, Chunlong Zhong, Yunlong Tan, Zhiren Wang, Jijun Wang, Xiaoping Wang, Longli Kang, Lu Lu, Xiangning Chen, Chiang-Shan R. Li and Xingguang Luo

**Table S1.** Significant expression correlation between *CHRNs* and dopaminergic and GABAergic receptor genes in ten human brain areas.

| **Brain** |  | **CHRNB3** | | **CHRNA6** | | **CHRNA5** | | **CHRNA3** | | **CHRNB4** | | **CHRNA4** | |
| --- | --- | --- | --- | --- | --- | --- | --- | --- | --- | --- | --- | --- | --- |
| **Area** | **Gene** | **r** | ***p*** | **r** | ***p*** | **r** | ***p*** | **r** | ***p*** | **r** | ***p*** | **r** | ***p*** |
| cerebellar cortex (CRBL) | DRD2 |  |  |  |  |  |  |  |  | 0.462 | 3.2 × 10^−8^ | 0.394 | 3.5 × 10^−6^ |
|  | DRD5 |  |  |  |  |  |  |  |  | 0.513 | 4.5 × 10^−10^ |  |  |
|  | TH |  |  |  |  |  |  |  |  | 0.577 | 6.5 × 10^−13^ | 0.414 | 9.5 × 10^−7^ |
|  | GABRA1 |  |  |  |  |  |  | 0.574 | 9.7 × 10^−13^ |  |  |  |  |
|  | GABRA2 |  |  |  |  | 0.461 | 3.5 × 10^−8^ |  |  |  |  |  |  |
|  | GABRA3 |  |  |  |  |  |  | 0.357 | 3.0 × 10^−5^ |  |  | 0.480 | 7.6 × 10^−9^ |
|  | GABRA4 |  |  |  |  | 0.401 | 2.3 × 10^−6^ |  |  |  |  | 0.389 | 4.9 × 10^−6^ |
|  | GABRA5 |  |  |  |  |  |  |  |  | 0.384 | 6.5 × 10^−6^ | 0.477 | 9.2 × 10^−9^ |
|  | GABRA6 |  |  |  |  |  |  | 0.513 | 4.3 × 10^−10^ |  |  |  |  |
|  | GABRB1 |  |  |  |  | 0.455 | 5.5 × 10^−8^ |  |  |  |  |  |  |
|  | GABRB2 |  |  |  |  | 0.371 | 1.4 × 10^−5^ | 0.400 | 2.5 × 10^−6^ |  |  |  |  |
|  | GABRB3 |  |  |  |  |  |  |  |  |  |  | 0.453 | 6.1 × 10^−8^ |
|  | GABRD |  |  |  |  |  |  | 0.432 | 2.9 × 10^−7^ |  |  |  |  |
|  | GABRG1 |  |  |  |  | 0.470 | 1.6 × 10^−8^ |  |  |  |  |  |  |
|  | GABRG2 |  |  |  |  |  |  | 0.563 | 3.2 × 10^−12^ |  |  |  |  |
|  | GABRG3 |  |  |  |  | 0.448 | 9.1 × 10^−8^ |  |  |  |  | 0.372 | 1.3 × 10^−5^ |
|  | GABRP |  |  |  |  |  |  |  |  | 0.406 | 1.6 × 10^−6^ |  |  |
|  | GABRQ |  |  |  |  |  |  |  |  | 0.426 | 4.4 × 10^−7^ | 0.571 | 1.3 × 10^−12^ |
| frontal cortex (FCTX) | DRD2 |  |  |  |  |  |  | 0.529 | 1.6 × 10^−10^ | 0.392 | 5.3 × 10^−6^ |  |  |
|  | DRD3 | −0.434 | 3.6 × 10^−7^ |  |  |  |  | 0.439 | 2.4 × 10^−7^ | 0.455 | 7.5 × 10^−8^ |  |  |
|  | DRD4 | −0.361 | 3.0 × 10^−5^ |  |  |  |  | 0.590 | 2.9 × 10^−13^ | 0.566 | 3.9 × 10^−12^ |  |  |
|  | DRD5 |  |  |  |  |  |  | 0.500 | 2.1 × 10^−9^ | 0.554 | 1.3 × 10^−11^ |  |  |
|  | TH |  |  |  |  |  |  | 0.526 | 2.1 × 10^−10^ | 0.618 | 9.8 × 10^−15^ |  |  |
|  | GABRA1 |  |  |  |  |  |  |  |  | −0.363 | 2.7 × 10^−5^ | 0.374 | 1.5 × 10^−5^ |
|  | GABRA2 |  |  |  |  |  |  | −0.430 | 4.4 × 10^−7^ | −0.369 | 1.9 × 10^−5^ |  |  |
|  | GABRA3 |  |  |  |  |  |  |  |  |  |  | 0.440 | 2.3 × 10^−7^ |
|  | GABRA4 | 0.399 | 3.5 × 10^−6^ |  |  |  |  | −0.398 | 3.6 × 10^−6^ | −0.374 | 1.5 × 10^−5^ | 0.391 | 5.3 × 10^−6^ |
|  | GABRA5 |  |  |  |  |  |  |  |  |  |  | 0.498 | 2.6 × 10^−9^ |
|  | GABRB1 | 0.391 | 5.5 × 10^−6^ |  |  |  |  | −0.521 | 3.3 × 10^−10^ | −0.487 | 6.2 × 10^−9^ |  |  |
|  | GABRB2 |  |  |  |  |  |  | −0.364 | 2.6 × 10^−5^ | −0.404 | 2.4 × 10^−6^ | 0.368 | 2.0 × 10^−5^ |
|  | GABRB3 |  |  |  |  |  |  |  |  |  |  | 0.406 | 2.2 × 10^−6^ |
|  | GABRD |  |  |  |  |  |  |  |  |  |  | 0.504 | 1.5 × 10^−9^ |
|  | GABRG3 | 0.385 | 7.9 × 10^−6^ |  |  |  |  |  |  | −0.396 | 4.1 × 10^−6^ | 0.483 | 9.2 × 10^−9^ |
|  | GABRP | −0.394 | 4.6 × 10^−6^ |  |  |  |  | 0.472 | 2.1 × 10^−8^ | 0.470 | 2.5 × 10^−8^ |  |  |
| hippocampus (HIPP) | DRD2 |  |  |  |  |  |  | 0.604 | 1.9 × 10^−13^ |  |  |  |  |
|  | DRD4 |  |  |  |  |  |  |  |  | 0.391 | 8.3 × 10^−6^ |  |  |
|  | DRD5 |  |  |  |  |  |  |  |  | 0.435 | 5.5 × 10^−7^ |  |  |
|  | GABRA1 |  |  |  |  |  |  |  |  |  |  | 0.476 | 3.0 × 10^−8^ |
|  | GABRA3 |  |  |  |  |  |  |  |  |  |  | 0.452 | 1.7 × 10^−7^ |
|  | GABRA4 |  |  |  |  |  |  |  |  |  |  | 0.424 | 1.2 × 10^−6^ |
|  | GABRB2 |  |  |  |  |  |  |  |  |  |  | 0.422 | 1.3 × 10^−6^ |
|  | GABRD |  |  |  |  |  |  |  |  |  |  | 0.393 | 7.5 × 10^−6^ |
|  | GABRG2 |  |  |  |  |  |  |  |  |  |  | 0.373 | 2.4 × 10^−5^ |
|  | GABRQ | −0.378 | 1.7 × 10^−5^ |  |  |  |  | −0.372 | 2.4 × 10^−5^ |  |  |  |  |
| medulla (MEDU) | GABRA1 |  |  |  |  |  |  |  |  |  |  | 0.462 | 1.2 × 10^−7^ |
|  | GABRA2 |  |  | 0.731 | 3.7 × 10^−21^ | 0.658 | 4.0 × 10^−16^ | 0.565 | 2.3 × 10^−11^ | 0.394 | 9.5 × 10^−6^ |  |  |
|  | GABRA3 |  |  | 0.599 | 5.9 × 10^−13^ | 0.563 | 2.6 × 10^−11^ |  |  |  |  |  |  |
|  | GABRA4 |  |  | 0.848 | 5.3 × 10^−34^ | 0.630 | 1.7 × 10^−14^ | 0.503 | 5.4 × 10^−9^ | 0.428 | 1.2 × 10^−6^ |  |  |
|  | GABRA5 |  |  | 0.556 | 5.2 × 10^−11^ | 0.449 | 3.0 × 10^−7^ | 0.424 | 1.5 × 10^−6^ |  |  |  |  |
|  | GABRB1 |  |  | 0.460 | 1.4 × 10^−7^ | 0.397 | 7.8 × 10^−6^ |  |  |  |  |  |  |
|  | GABRB2 |  |  |  |  |  |  |  |  |  |  | 0.433 | 8.5 × 10^−7^ |
|  | GABRB3 |  |  | 0.662 | 2.6 × 10^−16^ | 0.555 | 6.0 × 10^−11^ | 0.534 | 3.9 × 10^−10^ | 0.395 | 8.7 × 10^−6^ |  |  |
|  | GABRD |  |  |  |  |  |  |  |  |  |  | 0.413 | 3.1 × 10^−6^ |
|  | GABRE |  |  |  |  |  |  |  |  |  |  | 0.390 | 1.2 × 10^−5^ |
|  | GABRG1 |  |  | 0.508 | 3.7 × 10^−9^ | 0.470 | 7.1 × 10^−8^ | 0.390 | 1.2 × 10^−5^ |  |  |  |  |
|  | GABRG2 |  |  |  |  |  |  |  |  |  |  | 0.416 | 2.5 × 10^−6^ |
|  | GABRG3 |  |  | −0.372 | 3.1 × 10^−5^ |  |  |  |  |  |  | 0.422 | 1.7 × 10^−6^ |
|  | GABRQ |  |  |  |  |  |  |  |  |  |  | 0.394 | 9.1 × 10^−6^ |
| occipital cortex (OCTX) | DRD2 |  |  |  |  |  |  |  |  |  |  | 0.358 | 3.0 × 10^−5^ |
|  | DRD3 |  |  |  |  |  |  |  |  | 0.376 | 1.1 × 10^−5^ |  |  |
|  | DRD4 |  |  |  |  |  |  |  |  | 0.554 | 9.8 × 10^−12^ |  |  |
|  | TH |  |  |  |  |  |  |  |  | 0.461 | 3.9 × 10^−8^ |  |  |
|  | GABRA2 | 0.418 | 8.0 × 10^−7^ | 0.366 | 2.0 × 10^−5^ |  |  |  |  |  |  | 0.406 | 1.8 × 10^−6^ |
|  | GABRA3 | 0.429 | 3.8 × 10^−7^ | 0.453 | 7.0 × 10^−8^ |  |  |  |  |  |  | 0.573 | 1.3 × 10^−12^ |
|  | GABRA4 | 0.403 | 2.2 × 10^−6^ | 0.388 | 5.5 × 10^−6^ |  |  |  |  |  |  |  |  |
|  | GABRA5 | 0.457 | 5.3 × 10^−8^ | 0.427 | 4.4 × 10^−7^ |  |  |  |  |  |  | 0.541 | 3.7 × 10^−11^ |
|  | GABRB1 | 0.379 | 9.5 × 10^−6^ |  |  |  |  | −0.444 | 1.4 × 10^−7^ |  |  | 0.521 | 2.4 × 10^−10^ |
|  | GABRB3 | 0.429 | 3.9 × 10^−7^ | 0.449 | 9.7 × 10^−8^ |  |  |  |  |  |  | 0.442 | 1.6 × 10^−7^ |
|  | GABRG1 |  |  |  |  |  |  | −0.460 | 4.1 × 10^−8^ |  |  |  |  |
|  | GABRG2 | 0.368 | 1.8 × 10^−5^ |  |  |  |  |  |  |  |  |  |  |
| putamen (PUTM) | DRD1 |  |  | −0.682 | 5.9 × 10^−19^ |  |  |  |  |  |  |  |  |
|  | DRD2 |  |  | −0.558 | 6.3 × 10^−12^ |  |  |  |  |  |  |  |  |
|  | DRD3 |  |  | −0.433 | 3.0 × 10^−7^ |  |  |  |  |  |  |  |  |
|  | DRD4 |  |  |  |  |  |  |  |  | 0.441 | 1.7 × 10^−7^ |  |  |
|  | GABRA2 |  |  | −0.507 | 9.1 × 10^−10^ |  |  |  |  |  |  |  |  |
|  | GABRA3 |  |  |  |  |  |  |  |  |  |  | 0.522 | 2.2 × 10^−10^ |
|  | GABRA4 |  |  | −0.378 | 1.0 × 10^−5^ |  |  |  |  |  |  |  |  |
|  | GABRB1 | −0.361 | 2.6 × 10^−5^ | −0.450 | 8.7 × 10^−8^ |  |  |  |  |  |  |  |  |
|  | GABRB2 |  |  |  |  |  |  | 0.358 | 3.1 × 10^−5^ |  |  |  |  |
|  | GABRD |  |  |  |  |  |  | 0.357 | 3.2 × 10^−5^ |  |  |  |  |
|  | GABRG3 |  |  | −0.590 | 1.9 × 10^−13^ |  |  |  |  |  |  |  |  |
|  | GABRP | 0.366 | 2.0 × 10^−5^ |  |  |  |  |  |  | 0.359 | 2.9 × 10^−5^ |  |  |
|  | GABRQ |  |  |  |  |  |  |  |  |  |  | 0.433 | 3.0 × 10^−7^ |
| substantia nigra | DRD1 | −0.417 | 1.4 × 10^−5^ |  |  |  |  |  |  |  |  |  |  |
|  | DRD2 | 0.834 | 2.9 × 10^−27^ | 0.807 | 2.4 × 10^−24^ |  |  |  |  |  |  | 0.802 | 6.8 × 10^−24^ |
|  | DRD5 |  |  |  |  |  |  |  |  | 0.485 | 2.8 × 10^−7^ |  |  |
|  | TH | 0.920 | 3.9 × 10^−42^ | 0.902 | 7.4 × 10^−38^ |  |  |  |  |  |  | 0.909 | 2.5 × 10^−39^ |
|  | GABRA1 | 0.482 | 3.4 × 10^−7^ | 0.487 | 2.4 × 10^−7^ | 0.436 | 5.3 × 10^−6^ |  |  |  |  | 0.454 | 1.9 × 10^−6^ |
|  | GABRA3 | 0.590 | 8.1 × 10^−11^ | 0.617 | 6.1 × 10^−12^ | 0.464 | 1.0 × 10^−6^ |  |  |  |  | 0.625 | 2.7 × 10^−12^ |
|  | GABRA4 | 0.622 | 3.9 × 10^−12^ | 0.679 | 6.1 × 10^−15^ |  |  |  |  |  |  | 0.625 | 2.9 × 10^−12^ |
|  | GABRB1 | 0.735 | 2.1 × 10^−18^ | 0.725 | 9.5 × 10^−18^ | 0.401 | 3.3 × 10^−5^ |  |  |  |  | 0.649 | 2.2 × 10^−13^ |
|  | GABRB2 | 0.410 | 2.1 × 10^−5^ | 0.419 | 1.3 × 10^−5^ |  |  |  |  |  |  |  |  |
|  | GABRB3 | 0.417 | 1.4 × 10^−5^ | 0.471 | 6.7 × 10^−7^ |  |  |  |  |  |  | 0.503 | 8.4 × 10^−8^ |
|  | GABRG2 | 0.624 | 3.2 × 10^−12^ | 0.623 | 3.6 × 10^−12^ |  |  |  |  |  |  | 0.612 | 1.0 × 10^−11^ |
| temporal cortex (TCTX) | DRD1 |  |  | −0.375 | 2.6 × 10^−5^ |  |  | −0.521 | 1.2 × 10^−9^ |  |  |  |  |
|  | DRD2 | 0.514 | 2.2 × 10^−9^ | 0.536 | 3.5 × 10^−10^ |  |  | 0.376 | 2.5 × 10^−5^ |  |  | 0.493 | 1.3 × 10^−8^ |
|  | DRD4 |  |  |  |  |  |  | 0.488 | 1.8 × 10^−8^ | 0.552 | 7.9 × 10^−11^ |  |  |
|  | TH | 0.555 | 5.5 × 10^−11^ | 0.433 | 8.8 × 10^−7^ |  |  | 0.432 | 9.5 × 10^−7^ |  |  |  |  |
|  | GABRA2 |  |  |  |  |  |  | −0.482 | 2.8 × 10^−8^ |  |  |  |  |
|  | GABRA3 |  |  |  |  |  |  |  |  |  |  | 0.427 | 1.3 × 10^−6^ |
|  | GABRA4 |  |  |  |  |  |  | −0.435 | 7.5 × 10^−7^ | −0.394 | 9.1 × 10^−6^ |  |  |
|  | GABRB1 |  |  |  |  |  |  | −0.502 | 6.0 × 10^−9^ | −0.410 | 3.6 × 10^−6^ |  |  |
|  | GABRB2 |  |  |  |  |  |  | −0.376 | 2.5 × 10^−5^ | −0.405 | 5.0 × 10^−6^ |  |  |
|  | GABRB3 |  |  |  |  |  |  | −0.373 | 3.0 × 10^−5^ | −0.379 | 2.1 × 10^−5^ |  |  |
|  | GABRG2 |  |  |  |  |  |  | −0.373 | 2.9 × 10^−5^ | −0.385 | 1.5 × 10^−5^ |  |  |
|  | GABRG3 |  |  |  |  |  |  | −0.559 | 3.8 × 10^−11^ | −0.402 | 5.8 × 10^−6^ |  |  |
| thalamus (THAL) | DRD1 |  |  |  |  |  |  |  |  |  |  | −0.372 | 2.1 × 10^−5^ |
|  | DRD2 |  |  | 0.567 | 6.6 × 10^−12^ |  |  | 0.560 | 1.3 × 10^−11^ |  |  | 0.520 | 5.9 × 10^−10^ |
|  | DRD3 |  |  |  |  | −0.412 | 2.0 × 10^−6^ |  |  |  |  |  |  |
|  | DRD4 |  |  |  |  | −0.437 | 4.0 × 10^−7^ |  |  |  |  |  |  |
|  | DRD5 |  |  | −0.459 | 8.2 × 10^−8^ | −0.438 | 3.7 × 10^−7^ | −0.389 | 8.0 × 10^−6^ |  |  | −0.458 | 9.1 × 10^−8^ |
|  | GABRA1 |  |  | 0.778 | 2.3 × 10^−26^ | 0.545 | 6.1 × 10^−11^ | 0.773 | 6.8 × 10^−26^ |  |  | 0.808 | 8.5 × 10^−30^ |
|  | GABRA2 |  |  | 0.511 | 1.3 × 10^−9^ |  |  | 0.427 | 7.3 × 10^−7^ |  |  | 0.426 | 8.0 × 10^−7^ |
|  | GABRA3 |  |  | 0.751 | 1.1 × 10^−23^ | 0.557 | 1.8 × 10^−11^ | 0.828 | 1.7 × 10^−32^ |  |  | 0.867 | 8.7 × 10^−39^ |
|  | GABRA4 |  |  | 0.698 | 2.1 × 10^−19^ | 0.549 | 4.1 × 10^−11^ | 0.781 | 1.1 × 10^−26^ |  |  | 0.753 | 6.9 × 10^−24^ |
|  | GABRA5 |  |  | 0.746 | 2.6 × 10^−23^ | 0.562 | 1.1 × 10^−11^ | 0.833 | 3.9 × 10^−33^ |  |  | 0.844 | 8.9 × 10^−35^ |
|  | GABRB1 |  |  | 0.395 | 5.5 × 10^−6^ |  |  |  |  |  |  |  |  |
|  | GABRB2 |  |  | 0.767 | 3.2 × 10^−25^ | 0.546 | 5.2 × 10^−11^ | 0.809 | 6.0 × 10^−30^ |  |  | 0.836 | 1.3 × 10^−33^ |
|  | GABRB3 |  |  | 0.716 | 8.5 × 10^−21^ | 0.450 | 1.5 × 10^−7^ | 0.742 | 6.9 × 10^−23^ |  |  | 0.753 | 6.5 × 10^−24^ |
|  | GABRD |  |  | 0.624 | 1.0 × 10^−14^ | 0.551 | 3.3 × 10^−11^ | 0.799 | 1.1 × 10^−28^ |  |  | 0.831 | 6.7 × 10^−33^ |
|  | GABRE |  |  | 0.376 | 1.6 × 10^−5^ |  |  |  |  |  |  |  |  |
|  | GABRG2 |  |  | 0.743 | 5.2 × 10^−23^ | 0.499 | 3.6 × 10^−9^ | 0.737 | 1.7 × 10^−22^ |  |  | 0.768 | 2.2 × 10^−25^ |
|  | GABRQ |  |  | 0.550 | 3.7 × 10^−11^ | 0.423 | 9.7 × 10^−7^ | 0.506 | 2.0 × 10^−9^ |  |  | 0.595 | 3.3 × 10^−13^ |
|  | GABRR2 |  |  |  |  |  |  | 0.487 | 9.5 × 10^−9^ |  |  |  |  |
| WHMT | DRD5 | 0.390 | 4.2 × 10^−6^ |  |  |  |  | 0.398 | 2.4 × 10^−6^ | 0.474 | 1.1 × 10^−8^ |  |  |
|  | TH | 0.401 | 2.1 × 10^−6^ |  |  |  |  |  |  |  |  |  |  |
|  | GABRA4 | 0.477 | 8.3 × 10^−9^ | 0.488 | 3.5 × 10^−9^ |  |  | 0.367 | 1.6 × 10^−5^ |  |  |  |  |
|  | GABRD | 0.363 | 2.0 × 10^−5^ |  |  |  |  | 0.399 | 2.4 × 10^−6^ |  |  |  |  |
|  | GABRG2 | 0.373 | 1.2 × 10^−5^ |  |  |  |  |  |  |  |  |  |  |

Data came from the UK Brain Expression Consortium (UKBEC). The statistical significance level (α) =3.3 × 10^−5^. r, Pearson correlation coefficient. *p*, *p* values.

**Table S2.** Significant expression correlation between *CHRNs* and dopaminergic and GABAergic receptor genes in human frontal cortex.

|  | **CHRNA3** | | **CHRNA4** | | **CHRNB3** | |
| --- | --- | --- | --- | --- | --- | --- |
|  | **β** | ***p*** | **β** | ***p*** | **β** | ***p*** |
| DRD1 | −0.340 | 2.4 × 10^−8^ | 0.492 | 3.6 × 10^−9^ | 0.202 | 6.5 × 10^−10^ |
| DRD2 | 0.234 | 6.1 × 10^−16^ |  |  | −0.088 | 6.4 × 10^−7^ |
| DRD4 | 0.538 | 1.2 × 10^−9^ |  |  |  |  |
| TH | 0.749 | 3.8 × 10^−13^ |  |  |  |  |
| GABRA1 |  |  | 0.028 | 1.3 × 10^−13^ | 0.010 | 4.9 × 10^−10^ |
| GABRA2 |  |  |  |  | 0.027 | 2.7 × 10^−8^ |
| GABRA4 |  |  | 0.062 | 1.7 × 10^−12^ | 0.022 | 1.4 × 10^−9^ |
| GABRA5 |  |  | 0.092 | <2 × 10^−16^ | 0.028 | 7.1 × 10^−10^ |
| GABRB2 |  |  | 0.033 | 2.0 × 10^−9^ | 0.013 | 1.9 × 10^−9^ |
| GABRB3 |  |  | 0.059 | 3.3 × 10^−12^ | 0.021 | 4.9 × 10^−10^ |
| GABRG2 |  |  | 0.020 | 2.9 × 10^−11^ | 0.008 | 6.7 × 10^−10^ |
| GABRG3 |  |  | 0.082 | 2.2 × 10^−11^ | 0.028 | 4.8 × 10^−8^ |

Data came from Heinzen et al. 2008. The statistical significance level (α) =6.9 × 10^−7^. β, regression coefficient from generalized linear model (GLM).

| D | 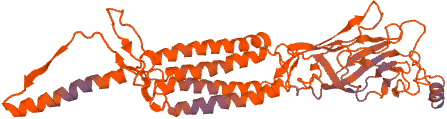 |
| --- | --- |
| N | 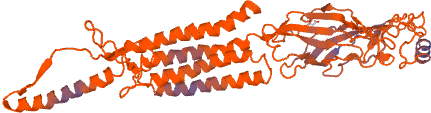 |

**Figure S1.** The tertiary structures of α5 nAChR altered by rs16969968 (D: Asp; N: Asn).
